# Climate change and mental health: a call to action to include mental health and psychosocial support services (MHPSS) in the Pakistan flood crisis

**DOI:** 10.1192/bji.2023.13

**Published:** 2023-08

**Authors:** Mehr Muhammad Adeel Riaz, Bismah Nayyer, Arush Lal, Faisal A. Nawaz, Ahsan Zil-e-Ali

**Affiliations:** 1 Department of Psychiatry and Behavioural Sciences, Punjab Medical College, Faisalabad, Pakistan. Email: adeelriaz369@gmail.com; 2 Department of Population Health Sciences, King's College London, London, UK; 3 Department of Health Policy, London School of Economics & Political Science, London, UK; 4 Department of Psychiatry, Al Amal Psychiatric Hospital, Al Aweer, Dubai, United Arab Emirates; 5 Heart and Vascular Institute, Pennsylvania State University, Hershey, Pennsylvania, USA

**Keywords:** Mental health and psychosocial support, trauma, mental health policy, flood crisis, Pakistan

## Abstract

The recent flood crisis in Pakistan has had significant impacts on the physical, mental and socioeconomic fabric of almost 33 million people. Floods in Pakistan are leading to a range of negative impacts on health and major disruptions to healthcare services. The lack of mental health and psychosocial support services (MHPSS) is a significant concern in rural areas of Pakistan in providing support to communities affected by floods. It is important for the government and mental health policymakers to work with academic coalitions and non-governmental organisations to replicate low-resource MHPSS models that will develop and advocate for effective, gender-sensitive mental healthcare throughout the country.

As the 27th Conference of the Parties to the United Nations Framework Convention on Climate Change (COP 27) concluded in November 2022, the recent flood crisis in Pakistan took the centre stage in Pakistan's advocacy efforts for climate justice on international platforms, resulting in the establishment of a ‘loss and damage fund’ for vulnerable countries.^1^ Pakistan recently co-chaired the international conference on Climate Resilient Pakistan along with the United Nations (UN) in Geneva.^2^ The conference served as an initiating effort for a systematic approach to aid Pakistan's recovery, by coordinating humanitarian aid and early recovery efforts to support sustainable long-term development in the country in line with the Resilient Recovery, Rehabilitation, and Reconstruction Framework (4RF) model proposed by the government.^2^ Pakistan is a lower-middle-income country, serving as one of the prime examples of the impacts of climate change on the physical, mental and socioeconomic fabric of vulnerable communities. The lack of mental health components in disaster relief plans highlights the need for responsible and conscious humanitarian action to alleviate the traumatic impact of floods on affected communities. Despite contributing less than 1% of global greenhouse gas emissions, Pakistan continues to climb up the climate vulnerability ladder, with the German Watch Index ranking Pakistan among the top 10 climate-vulnerable countries.^2^

## The 2022 floods

Pakistan has experienced the worst national disaster in its history owing to the June 2022 floods, which have also been declared a major health emergency.^3^ The disaster has affected 33 million people, resulting in 15 million displaced people, 12 867 injuries and 1700 deaths.^3^ The destruction of over 2000 health facilities and disruption of healthcare services has left 8.2 million people in flood-affected areas without urgent healthcare services.^3^ Floods have a substantial mental health impact, mediated by physical trauma and economic insecurity, increased gender-based violence, domestic abuse and bereavement. The stressors resulting from financial insecurity intersect with direct flood impacts to increase the prevalence of mental health conditions such as anxiety, complicated grief, depression and substance misuse.^4^ Consequences of disastrous climate events range from post-traumatic stress disorder due to the experience of flooding to the impacts of forced displacement on climate refugees.^4^ Similar to previous natural disasters in Pakistan, such as the 2005 earthquakes in the northern areas, the absence of mental health and psychosocial support services (MHPSS) has been observed.^5^ The population has experienced a significant increase in mental health conditions, as evidenced by data indicating a surge in depression following flooding. In fact, clinical diagnoses of major depressive disorder were reported in 61% of the population studied.^6^ The Ministry of Planning Development & Special Initiatives (MoPDSI) reports that 1 in 5 people in flood-affected areas will need mental healthcare services.^7^ According to Global Burden of Disease data, in 2019 approximately 25 million (11%) of the country's population suffered from mental health conditions.^8^

In Pakistan, only 500 psychiatrists^9^ serve the population of approximately 220 million, with almost all of them concentrated in urban areas. Thus, this shortage is particularly pronounced in rural areas, where most people affected by recent floods live. Research conducted by the UN and the MoPDSI found that among the 80 districts most severely affected by the disaster, approximately 83% of flood-affected districts in Balochistan, 56% in Sindh, 70% in Khyber Pakhtunkhwa, 66% in Punjab and 33% in Gilgit-Baltistan do not have a single psychiatrist.^10^ In many cases, these communities are low-income and are unaware of or unwilling to address psychological impacts.^10^ The baseline assessment of the initial mental health state in flood-affected regions of Pakistan indicates a surge in the prevalence of depression, anxiety and stress. Out of the individuals evaluated, 38%, 20% and 43% respectively were diagnosed with these mental health conditions directly attributed to the flood disaster.^11^

## Our call to action

In the wake of the flood disaster in Pakistan, it is important for the government and mental health policymakers, along with academic coalitions and non-governmental organisations (NGOs), to develop and advocate for fit-for-purpose techniques and services provided by mental health professionals to deliver gender-sensitive mental healthcare. The use of ecotherapy and ecopsychology techniques in clinical work can be helpful in supporting patients and helping them gain insight during the therapeutic process. It is also important to strengthen the capacity of mental health professionals by adapting and changing their clinical training and approaches to adopt a non-pathologising approach in treating strong emotions experienced during interactions with affected communities.

Furthermore, indoor clinical rounds can be repurposed to provide integrated patient care and space to interview patients who come from flood-affected areas to better understand their emotions and help address acute feelings of hopelessness and anxiety. Based on the emergency MHPSS model^10^ developed by the MoPDSI after the 2014 terrorist attack on the Army Public School in Peshawar, there has been increasing evidence of innovative digital models for MHPSS, focusing on providing culturally competent telemedicine services to patients.^8^ There is a dire need to establish community-based strategies to adopt task-shifting of MHPSS. The intervention may focus on integrating care at the primary healthcare level by increasing the capacity of non-specialists, including community representatives, primary healthcare providers, young mental health advocates, teachers, emergency responders and counsellors, to manage common mental health issues and recognise individuals who require professional support provided by district-level tertiary care specialists.

In this call to action, we urge mental health professionals and policymakers in Pakistan to align their efforts with established programmes that integrate MHPSS into disaster risk reduction strategies, as seen in low- and middle-income countries:

- initiate a multi-stakeholder dialogue on climate change and mental health by inviting national and international climate change advocates and experts to prioritise the provision of MHPSS with a gender-responsive approach
- develop and integrate climate change modules within the training curriculum for mental health professionals working in current and future climate change catastrophes
- include telepsychiatry in disaster relief packages along with essential health services delivery.

## Data Availability

Data availability is not applicable to this article as no new data were created or analysed in this study.
